# Genomic case report of a low grade bladder tumor metastasis to lung

**DOI:** 10.1186/s12894-018-0386-8

**Published:** 2018-09-03

**Authors:** Marvin J. Van Every, Garrett Dancik, Venki Paramesh, Grzegorz T. Gurda, David R. Meier, Steven E. Cash, Craig S. Richmond, Sunny Guin

**Affiliations:** 1 Department of Urology, Gundersen Health System, 1900 South Ave, La Crosse, WI 54601 USA; 2 Department of Mathematics and Computer Science, Eastern Connecticut State University, 83 Windham Street, Willimantic, CT 06226 USA; 3 Department of Cardiothoracic Surgery, Gundersen Health System, 1900 South Ave, La Crosse, WI 54601 USA; 4 Department of Pathology, Gundersen Health System, 1900 South Ave, La Crosse, WI 54601 USA; 5 Gundersen Medical Foundation, 1300 Badger Street, La Crosse, WI 54601 USA

**Keywords:** Bladder cancer, Lung metastasis, MTOR, KMT2D, RXRA, Exome sequencing

## Abstract

**Background:**

We present a rare case where distant metastasis of a low grade bladder tumor was observed. We carried out detailed genomic analysis and cell based experiments on patient tumor samples to study tumor evolution, possible cause of disease and provide personalized treatment strategies.

**Case presentation:**

A man with a smoking history was diagnosed with a low-grade urothelial carcinoma of the bladder and a concurrent high-grade upper urinary tract tumor. Seven years later he had a lung metastasis. We carried out exome sequencing on all the patient’s tumors and peripheral blood (germline) to identify somatic variants. We constructed a phylogenetic tree to capture how the tumors are related and to identify somatic changes important for metastasis. Although distant metastasis of low-grade bladder tumor is rare, the somatic variants in the tumors and the phylogenetic tree showed that the metastasized tumor had a mutational profile most similar to the low grade urothelial carcinoma. The primary and the metastatic tumors shared several important mutations, including in the *KMT2D* and the *RXRA* genes. The metastatic tumor also had an activating *MTOR* mutation, which may be important for tumor metastasis. We developed a mutational signature to understand the biologic processes responsible for tumor development. The mutational signature suggests that the tumor mutations are associated with tobacco carcinogen exposure, which is concordant with the patient’s smoking history. We cultured cells from the lung metastasis to examine proliferation and signaling mechanisms in response to treatment. The mTOR inhibitor Everolimus inhibited downstream mTOR signaling and induced cytotoxicity in the metastatic tumor cells.

**Conclusion:**

We used genomic analysis to examine a rare case of low grade bladder tumor metastasis to distant organ (lung). Our analysis also revealed exposure to carcinogens found is tobacco as a possible cause in tumor development. We further validated that the patient might benefit from mTOR inhibition as a potential salvage therapy in an adjuvant or recurrent disease setting.

**Electronic supplementary material:**

The online version of this article (10.1186/s12894-018-0386-8) contains supplementary material, which is available to authorized users.

## Background

Bladder cancer is the fourth most common cancer in men, with an estimated 79,030 new cases and 16,870 deaths expected in the United States in 2017 [1]. Unlike many other cancers, bladder cancer’s mortality rates and treatment options have changed very little in the last 30 years [1, 2]. Twenty-five percent of bladder cancers are muscle-invasive and life-threatening at diagnosis [3]. Non–muscle-invasive bladder cancers can recur [4], but they rarely metastasize to distant organs [4].

We present a genomic case report of a man diagnosed with low-grade bladder cancer and a separate, concurrent high-grade cancer of the upper urinary tract in 2009. The tumors were surgically removed. In 2016, the patient had a lung metastasis that, on pathology, resembled the low-grade bladder tumor that had been removed in 2009. Low-grade bladder tumors rarely metastasize to distant organs, so we used exome sequencing on the patient’s three tumor biopsy specimens (from 2009 and 2016) to investigate the relationship between the metastasis and the initial low-grade and high-grade tumors, to study tumor progression, and to identify therapeutic vulnerabilities. We also carried out primary culture of the patient’s lung cancer cells to study drug response.

## Case presentation

In July of 2009, a 56-year-old man with a 40 pack-year smoking history presented with a low-grade papillary urothelial (transitional cell) carcinoma at the right ureteral orifice (primary bladder tumor). He also had a high-grade urothelial carcinoma of the renal pelvis with focal squamous differentiation and extensive renal parenchymal involvement. His right ureter was filled with tumor but did not show intramuscular invasion. Venous and lymphatic invasion of this tumor was absent. The patient first underwent transurethral resection of the bladder tumor (TURBT) of right ureteral orifice for the bladder carcinoma and underwent an ureteroscopic resection of the right ureter. The final pathology report characterized the bladder tumor as low-grade, non-invasive transitional cell carcinoma, and the ureteral resection demonstrated low-grade transitional cell carcinoma. In August 2009 a month later, the patient’s upper urinary tract tumor was removed by hand-assisted laproscopic neproureterectomy. The final pathology on this tumor was pT3 pN0 with negative margins. The patient then underwent an intense course of 6 rounds of bacillus Calmette-Guérin (BCG) treatment in an adjuvant setting, followed by maintenance BCG treatment for 3 years.

In 2016 the patient returned with a lung tumor that, on pathologic evaluation, resembled the low grade right ureteral orifice bladder tumor (transitional cell) from July 2009. The lung tumor was surgically removed. Because low-grade bladder tumors rarely metastasize to distant organs, we consented the patient for an Institutional Review Board–approved research study to investigate the origin of the lung metastasis and also to identify genetic changes that could represent a therapeutic target for any future recurrence or metastasis.

**Fig. 1:**
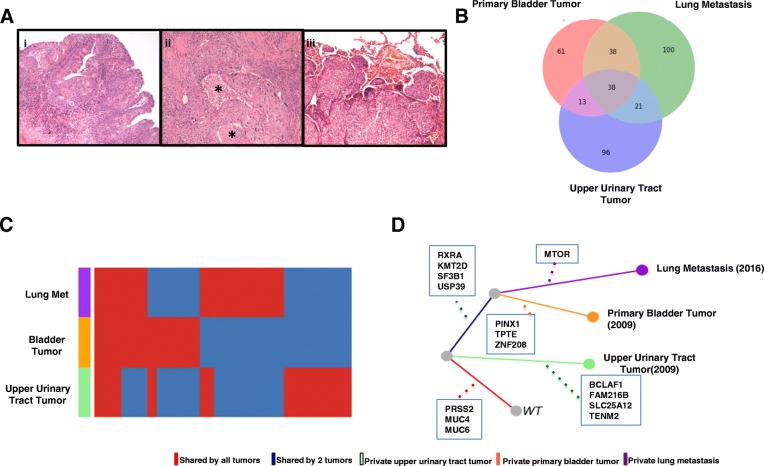
**a** Hematoxylin and eosin stain of biopsy specimens from **i)** primary bladder tumor, **ii)** upper urinary tract tumor, and **iii)** lung metastasis. The high-grade tumor shows marked cytologic atypia and central necrosis (indicated by asterisks) not seen within the other tumors. **b, c** Venn diagram and heat map showing variant overlaps among the three tumors. **d** Phylogenetic tree showing the evolutionary relationship of the three tumors

Hematoxylin and eosin (H&E) review of the formalin-fixed paraffin-embedded

(FFPE) tumor biopsy specimens showed more than 70% tumor tissue within all the samples sent for exome sequencing (Fig. 1a). The identified variants are listed in Additional file 1: Table S1, Additional file 2: Table S2, Additional file 3: Table S3, Additional file 4: Table S4. We focused on missense and nonsense somatic mutations present in the three tumor samples. Multiple variants were shared by these three tumors, and others were unique to each individual tumor, as shown by the Venn diagram and heat map (Fig. 1b, c).

Phylogenetic analysis indicates that the lung metastasis and primary bladder tumor are most closely related, and that the upper urinary tract tumor may have developed first (the distances between normal tissue and upper urinary tract tumor and normal tissue and primary bladder tumor are very similar, 168 gain/loss of single nucleotide variants (SNVs) versus 171 (Fig. 1d, Additional file 5: Table S5). No mutations in a known oncogene or tumor suppressor gene are shared by all three tumors; however, the primary bladder tumor and the lung metastasis share known oncogenic mutations frequently found in bladder tumors, such as mutations in the *KMT2D* and *RXRA* genes. The mutational signatures, histomorphology, and distinct anatomic sites indicate that the upper urinary tract tumor and the primary bladder tumor likely are unrelated. Conversely, based on mutational profile and our model of tumor evolution, the primary bladder tumor and the lung metastasis may be related.

With evidence that the lung metastasis may be derived from the primary bladder tumor, we analyzed the somatic variants present in the primary bladder tumor and the lung metastasis that might be responsible for tumor initiation and progression. Table 1 lists some of the important variants shared between the primary bladder tumor and the lung metastasis and also variants that are unique to the lung metastasis. The primary and the metastatic tumor have mutations in the *KMT2D* and *RXRA* genes. *KMT2D* encodes the protein histone-lysine N-methyltransferase 2D which is a tumor suppressor [5, 6]. *KMT2D* is mutated in 28% of bladder tumors [7]. *RXRA*, which encodes retinoid X receptor alpha (RXR-alpha), is mutated in 10% of bladder tumors [7]. The RXRA S427F mutation present in these patient tumors is a hotspot mutation that predominantly occurs in urothelial tumors [7–10]. Initial studies show that this particular *RXRA* mutation regulates lipid metabolism via peroxisome proliferator-activated receptor gamma (PPARG) activation [8]. Among the mutations unique to the lung metastasis, a clinically actionable, activating mutation in mTOR (C1483F) was identified. This particular *MTOR* mutation is also present in the primary bladder tumor (Table 1), but at a very low frequency (1%). This C1483F mTOR mutation has been shown to activate mTOR downstream signaling via phosphorylation of p70-S6K and 4E-BP1 [11]. Development or selection of a subpopulation of cells with this activating *MTOR* mutation may be the driving event for lung metastasis within the primary bladder tumor.

**Table 1:** List of a Few Important Variants Shared Between Primary Bladder Tumor And Lung Metastasis And Variants Unique to the Lung Metastasis

							Primary Bladder Tumor		Lung Metastasis		
	Chromosome	Position	Gene	AA Change	Ref	Alt	VAF	Read Depth	VAF	Read Depth	Altered in Bladder Cancer
Shared Between Primary Bladder Tumor and Lung Metastasis	12	49,420,607	*KMT2D*	R5048C	G	A	37%	169	30%	245	28%
	9	137,328,351	*RXRA*	S427F	C	T	48%	136	28%	184	10%
	2	198,267,350	*SF3B1*	Q669H	T	A	43%	212	41%	153	6%
	2	85,868,233	*USP39*	F473 L	C	A	27%	112	34%	108	4%
	12	2,566,842	*CACNA1C*	R243C	C	T	29%	69	30%	204	6%
	X	41,204,468	*DDX3X*	D354G	A	G	79%	179	53%	104	4%
	4	183,676,006	*TENM3*	R1496W	C	T	46%	108	26%	172	8%
Unique to Lung Metastasis	1	11,217,230	*MTOR*	C1483F	C	A	1%	160	19%	172	2.4%
	6	89,616,132	*RNGTT*	R132H	C	T	0%	128	22%	82	2.4%
	8	77,761,863	*ZFHX4*	V1254A	T	C	0%	187	22%	154	18%

*Ref* reference sequence, *Alt* alternate sequence, *VAF* variant allele frequency

**Fig. 2:**
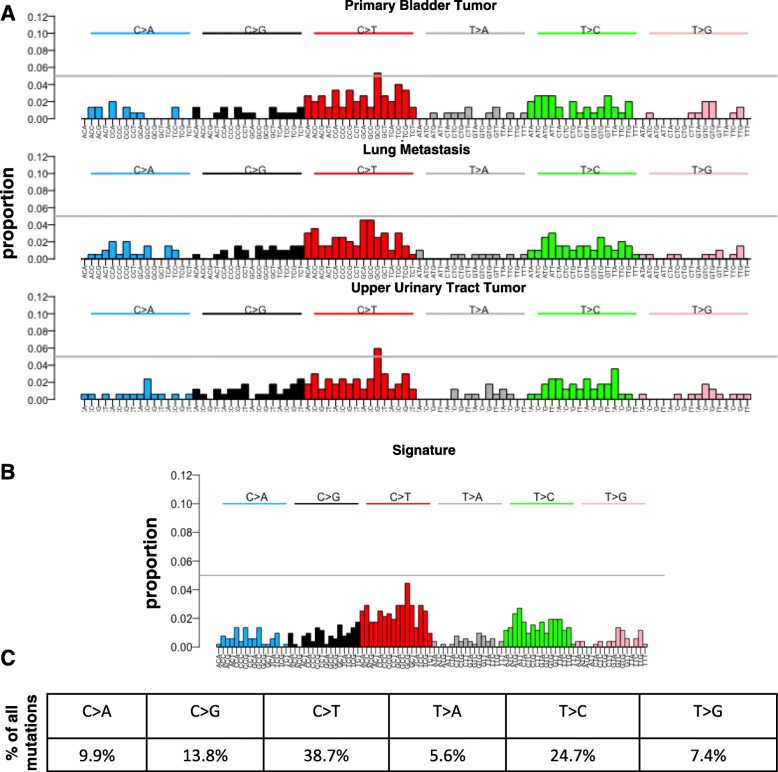
**a** Substitution patterns in the upper urinary tract tumor, primary bladder tumor, and lung metastasis isolated from the same patient. Substitutions are categorized according to the pyrimidine of the mutated base pair, for all possible trinucleotides that include the mutated base along with each neighboring base on the 5′ and 3′ ends. **b** Substitution signature for upper urinary tract tumor, primary bladder tumor, and lung metastasis isolated from the same patient. Proportion of substitutions as categorized according to the pyrimidine of the mutated base pair, for all possible trinucleotides that include the mutated base along with each neighboring base on the 5′ and 3′ ends. **c** Relative frequency table showing the percentages of each substitution across all samples (516 substitutions)

We carefully examined the mutations present in the patient tumors based on the base substitutions C > A, C > G, C > T, T > A, T > C, T > G to identify how the patient tumors correlate with known mutational signatures representative of various biological processes. Figure 2a shows the mutational landscape in all the three tumors. More C > T and T > C mutations were found in the three tumors. Next we developed a mutational signature for the patient by combining all the mutations present in these three tumors (Fig. 2b). This mutational signature is characterized by predominantly C > T and T > C mutations (Fig. 2c). This patient’s mutational signature resembles published mutational Signature 1A/B and Signature 5 [12]. Mutational signature 1A/B is related to the relatively elevated rate of spontaneous deamination of 5-methyl-cytosine, which results in C > T transitions and which predominantly occurs at NpCpG trinucleotides [12]. Signature 1A/B exhibits strong positive correlations with age in majority of cancers [12]. Signature 5, characterized by C > T and T > C mutations, is caused by tobacco carcinogens [12]. Our patient had a 40 pack-year smoking history, which suggests that tobacco use played a role in initiation of his tumors.

**Fig. 3:**
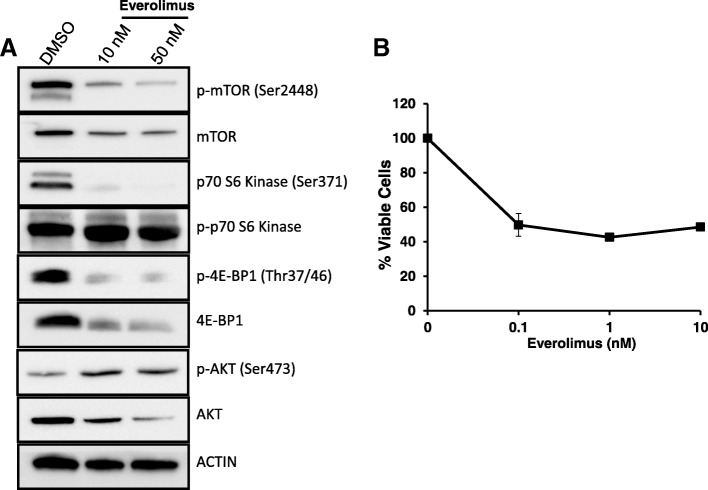
**a** Cells from the lung metastasis were treated with Everolimus at 10 and 50 nM concentrations for 24 h. The cells were lysed, and Western blots were carried out for proteins involved in mammalian target of rapamycin (mTOR) signaling. **b** 10^3^ cells were plated in triplicates in 96-well plate followed by treatment with increasing concentrations of everolimus. Cytotoxicity assay was carried out after 72-h drug treatment as described in Materials and Methods

Using the Drug Gene Interaction Database [13], we identified candidate drugs targeting 12 of the 100 genes with SNVs in the lung tumor (Additional file 6: Table S6). Several FDA-approved anti-cancer therapies were identified, including the *RXRA* agonist bexarotene and mTOR inhibitors, such as everolimus. We note that this analysis does not consider whether the variant is activating or deleterious, and all candidate therapies need to be evaluated. Primary culture of the lung metastasis was established in the laboratory. Since the lung metastasis has an activating *MTOR* mutation, we treated these cells with mTOR inhibitor everolimus at two concentrations (10 and 50 nM). The treatment showed a marked inhibition of mTOR activity and downstream signaling via two of its effectors, p70 S6K and 4E-BP1, at both concentrations (Fig. 3a); however, AKT activity increased with everolimus treatment (Fig. 3a). AKT can function both upstream and downstream of mTOR, but an increase in AKT activity could be a mechanism of resistance to the mTOR inhibitor.

Cytotoxicity study showed that at very low concentration (0.1 nM) everolimus reduces viability of these cells by about 60% (Fig. 3b), but even at a high concentration 40% of cells remain viable, indicating a cell population resistant to the drug.

## Discussion

We present a genomic case report of rare distant metastasis of a low grade bladder tumor. Phylogenetic analysis revealed that the primary low grade bladder tumor and the lung tumor are more closely related, and shared several known oncogenic mutations frequently observed in bladder tumors. Both these tumors presented with *KMT2D* and *RXRA* gene mutations. *KMT2D* is a known tumor suppressor that is mutated in a quarter of bladder tumors [5–7] and also regulates gene transcription [6]. We speculate that the loss of function of this tumor suppressor (*KMT2D* mutation seen here is likely inactivating) is an important driver for these tumors. Another important variant present in both these tumors is a mutation in the *RXRA* gene. The S427F RXRA mutation predominantly occurs in bladder tumors [9, 10]. Preliminary studies have shown that this particular *RXRA* mutation regulates *RXRA* and PPARG interaction [8]. There is increased activation of PPARG in tumors carrying this particular *RXRA* mutation, which drives lipid metabolism [7, 8]. We believe that RXRA S427F mutation may play an important role in tumor initiation and progression. Although outside the scope of this study, the role of this *RXRA* mutation in bladder cancer should be characterized in detail as another potential therapeutic avenue for patients with advanced bladder cancer.

The lung metastasis has an activating mutation in the *MTOR* gene (C1483F). This particular C1483F mutation was presented with a variant allele frequency (VAF) of 19% (of 172 reads) in the metastatic tumor and has a VAF of 1% (of 160 reads) in the primary bladder tumor. This indicates that a small population of cells in the primary tumor probably developed this particular *MTOR* mutation and this clonal population likely migrated and was able to seed in the lung. Thus, we think that the activating *MTOR* mutation could be one of the important drivers that lead to distant metastasis, perhaps thru evolution of a subclone of the low-grade bladder tumor. Treating the lung metastasis with everolimus showed a marked decrease in p70 S6K and 4E-BP1 activity, but a concurrent increase in activated AKT. Cytotoxicity assay showed that 60% of these cells are sensitive to everolimus treatment.t. We speculate that increase in AKT activity with the drug treatment could be a mechanism of resistance to mTOR inhibition and could explain the 40% viable cells post-treatment.

Finally, we tried to understand whether the mutations present in these tumors represent a known oncogenic process. The mutational signature in this patient was dominated by predominantly C > T and T > C mutations—the hallmark of a mutational signature caused by tobacco carcinogens [12]—suggesting that our patient’s tobacco use was perhaps responsible for his tumors.

We also tested whether the lung metastasis had any therapeutic vulnerability. We show that the metastasized tumor is vulnerable to mTOR inhibition because it carries an activating *MTOR* mutation. However if the patient has a recurrence of the lung metastasis and physicians choose to treat him with an mTOR inhibitor, they should take into account a possible mechanism of resistance driven by activated AKT. Thus, we believe that in case of another metastasis or a local recurrence, this patient may benefit from a combination of mTOR inhibitor and a conventional standard-of-care chemotherapy.

## Conclusion

Here we used genomic analysis to study cancer evolution and present a rare case where a low grade bladder tumor metastasis to the lung. We used phylogenetic analysis to prove that the low grade bladder tumor and the lung metastasis are closely related. We also conclude that this patient may benefit from an mTOR inhibitor in case of disease recurrence since his metastatic disease carries an activating mTOR mutation and cancer cells cultured from his lung tumor showed vulnerability to mTOR inhibition. This case report points to the importance of genomic analysis of patient tumors to understand tumor biology, evolution and for personalized patient care.

## Materials and methods

### Sample preparation for exome sequencing

Formalin-fixed paraffin-embedded (FFPE) tumor biopsy blocks from the primary bladder tumor (2009), high-grade upper urinary tract tumor (2009), and the lung metastasis (2016) were cut and stained with hematoxylin and eosin (H&E) staining using standard protocol [14] to identify the tumor-rich regions. gDNA was isolated from the tumor-rich regions of the biopsies using Qiagen QIAamp® DNA FFPE Tissue kit, per standard manufacturer instruction. gDNA was also isolated from patient blood to detect germline genetic changes using Qiagen QIAamp® DNA Blood Mini kit. Exome sequencing and variant calling was carried out at BGI Sequencing Services.

### Data analysis (exome sequencing, phylogenetic tree, substitution patterns and mutational signature, and druggable genes)

Somatic variants were identified by removing those present in the patient’s blood, and then filtered to remove those observed ≥0.1% in the 1000 Genomes and Exome Sequencing Projects. Downstream analyses were limited to non-silent variants and indels in the coding region of the gene.

Phylogenetic trees were constructed based on the presence or absence of somatic coding variants, using the parsimony ratchet method [15] as implemented in the Bioconductor package *phangorn* [16], version 2.2.0. Branch lengths were calculated using the ACCelerated TRANsformation criteria (*acctran* function).

Substitution patterns are categorized based on the mutated pyrimidine (C or T) or the complementary pyrimidine partner of the mutated base (for substitutions involving A or G), yielding the following mutations: C > A, C > G, C > T, T > A, T > C, T > G. The immediate 5′ and 3′ bases are included, for example, ACA > AAA, to yield 96 possible trinucleotide substitutions. The identification of mutation signatures was carried out using a previously published computational framework implemented in MATLAB [17]. This approach identifies mutation patterns (i.e., mutation signatures) that explain observed mutations across samples.

Druggable genes were identified by querying genes of interest against the Drug Gene Interaction Database [13] with the results “summarized by gene.” If > 3 drugs were identified for a specific gene, the top 3 drugs were reported.

### Primary culture of lung metastasized tumor

Tumor tissue from the surgically removed lung metastasis was collected using an IRB-approved protocol. This tissue was first digested with collagenase/hyaluronidase (Stemcell Technologies) at 37 °C for 3 h. This was followed by Accutase (Stemcell Technologies) digestion for 30 min at 37 °C. After Accutase digestion cells were filtered using a 40 μm cell strainer. The cells were suspended in HBSS (Thermo Fisher Scientific) with 2%FBS (Stemcell Technologies), 10 μM ROCK inhibitor Y-27632 (Stemcell Technologies). Epithelial cells were isolated using Human EpCAM Positive Selection Kit (Stemcell Technologies) using standard manufacturer protocol. The isolated epithelial cells were cultured in Hepatocyte Medium (Stemcell Technologies) with 10 ng/ml EGF (Stemcell Technologies), 5% heat-inactivated charcoal stripped FBS (Stemcell Technologies), Glutamax (Thermo Fisher Scientific), 5% Matrigel (Thermo Fisher Scientific), and 10 μM ROCK inhibitor Y-27632 [18].

### Western blot

Primary cells were treated with mTOR inhibitor everolimus (Cell Signaling) at 10 and 50 nM concentration for 24 h. The cells were lysed, followed by Western blot analysis for phosphorylated and total mTOR, p70 S6K, 4E-BP1, and AKT. Actin was used as housekeeping control. All primary antibodies were from Cell Signaling. HRP (Cell Signaling) labeled mouse or rabbit secondary antibodies were used, followed by chemiluminescence using ECL (Pierce, Rockford, IL).

### Cytotoxicity study

Cells were plated in 96 well plates (10^3^ cells/well) in triplicates and treated with different concentrations of everolimus or vehicle control. After 72 h of drug treatment, cell viability was measured using CyQUANT® Cell Proliferation Assay (Thermo Fisher Scientific) according to manufacturer instructions.

## Additional files

Additional file 1: **Table S1.** Single nucleotide variants detected in primary bladder tumor. (CSV 13537 kb)

Additional file 2: **Table S2.** Single nucleotide variants detected in upper urinary tract tumor. (CSV 13751 kb)

Additional file 3: **Table S3.** Single nucleotide variants detected in lung metastasis. (CSV 13575 kb)

Additional file 4: **Table S4.** Single nucleotide variants detected in peripheral blood. (CSV 13764 kb)

Additional file 5: **Table S5.** Single nucleotide variants shared between and unique to the three tumors. (XLSX 149 kb)

Additional file 6: **Table S6.** Candidate drug targets of genes with SNVs in the lung tumor. (XLSX 37 kb)

## Abbreviations

BCG: bacillus Calmette-Guerin
FFPE: Formalin-fixed paraffin-embedded
H&E: Hematoxylin and eosin
